# Sarcoid of the Upper Humerus Found Incidentally on MR Images Obtained for Work-Up of Rotator Cuff Tear Where Compromised Tissue Quality Was a Concern for Surgical Success

**DOI:** 10.1155/2018/3579527

**Published:** 2018-06-10

**Authors:** Tanner R. Henrie, John G. Skedros

**Affiliations:** ^1^Tulane University School of Medicine, New Orleans, LA, USA; ^2^The University of Utah Department of Orthopaedic Surgery, Salt Lake City, UT, USA; ^3^Utah Orthopaedic Specialists, Salt Lake City, UT, USA; ^4^Intermountain Medical Center, Salt Lake City, UT, USA

## Abstract

Sarcoidosis is an idiopathic systemic inflammatory disorder characterized histologically by noncaseating granulomas. The pathogenesis likely includes genetic, immunologic, and environmental factors. The lungs, skin, and eyes are most commonly affected. Although bone involvement is possible, sarcoidosis of the humerus is rare, with few cases reported. Furthermore, we are unaware of any reports of sarcoidosis of the upper humerus with a coexisting rotator cuff tear. We report the case of a 50-year-old female with sarcoidosis of the humerus and a coexisting tear of the supraspinatus tendon. Her medical history includes type 2 diabetes, depression, and fatigue. She had chronic shoulder pain that worsened after her dog jerked on the leash. Radiographs were grossly normal. Subsequent magnetic resonance imaging (MRI) demonstrated a possible small full-thickness rotator cuff tear. Multiple rounded lesions were also noted within the proximal humerus. A biopsy demonstrated noncaseating granulomas, confirming the diagnosis of sarcoidosis. There was concern that her sarcoid lesions would compromise bone quality, limiting options for surgical repair of her rotator cuff tear. However, it was determined that her lesion did not involve cortical bone, and surgery was performed. During surgery, the supraspinatus tendon was found to be partially torn and was treated with arthroscopic debridement and acromioplasty. An excellent result was ultimately achieved after her rheumatologist started adalimumab injections. This case demonstrates that there can be a rare incidental finding of osseous sarcoid lesions in the upper humerus where the bone might be compromised in the location of a planned rotator cuff repair.

## 1. Introduction

Grunewald et al. [1] described sarcoidosis as a systemic inflammatory disorder characterized by tissue infiltration by mononuclear phagocytes and lymphocytes with associated noncaseating granuloma formation. The proposed pathogenesis includes a combination of genetic, immunologic, and environmental factors [2]. Although the etiology of sarcoidosis remains uncertain, there is a growing body of evidence suggesting a relationship between sarcoidosis and an infectious process [2, 3].

Lung involvement is the most common manifestation of sarcoidosis, being present in over 90% of patients [4]. The skin and eyes are also commonly affected [5, 6]. Bone involvement in sarcoidosis is uncommon, affecting roughly 1–13% of patients [5, 6]. However, bone involvement may be underestimated, as approximately 38% of patients with sarcoidosis demonstrate radionuclide bone scan abnormalities [7]. Bone lesions may cause pain, but are typically asymptomatic [8]. The more commonly affected bones are small tubular bones of the appendicular skeleton, while axial bones are typically spared [6]. Osseous sarcoid may affect the hands, feet, tibia, skull, and vertebrae, among other bones. It typically causes nonspecific osteolytic lesions that can mimic neoplasms (e.g., osteoblastoma, metastases, or multiple myeloma), osteomyelitis, or bone cysts [5, 8–12].

Sarcoidosis of the humerus is rare and only a few cases have been reported [7–9, 11, 13–15]. We report a rare case where sarcoidosis of the upper humerus was found incidentally with an MRI scan that was obtained for work-up of a rotator cuff tear.

## 2. Case Presentation

A 50-year-old female (BMI = 35) presented to our clinic in Salt Lake City, Utah, USA, with a chief complaint of right shoulder pain. She has a history of non-insulin-dependent diabetes, hypertension, anxiety, depression, and fatigue. Her medications included hydroxyzine for anxiety, ibuprofen for joint pains, lisinopril for hypertension, and pioglitazone tablets and liraglutide (Victoza®; Novo Nordisk A/S, Bagsvaerd, Denmark) subcutaneous injections for diabetes. She had a several-year history of intermittent low-grade right shoulder pain that was attributed to subacromial bursitis. This had been treated with subacromial corticosteroid injections and physical therapy, which only gave moderate pain relief.

**Figure 1:**
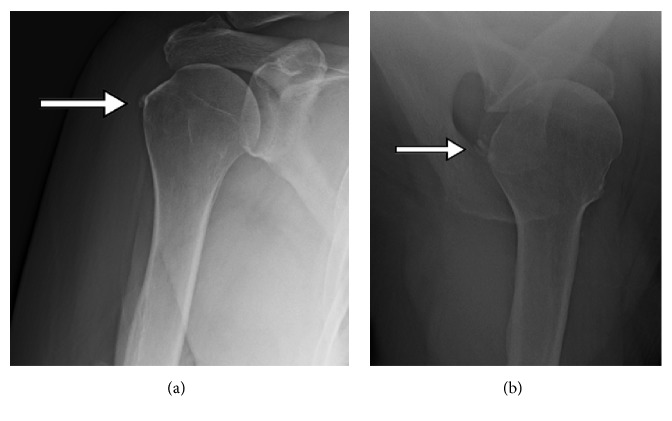
Radiographs with no distinct bone lesions noted. However, subtle decrease in trabecular bone density adjacent to the greater tuberosity is noted in (a) near the arrow tip. The arrows also indicate mild calcific tendinitis.

**Figure 2:**
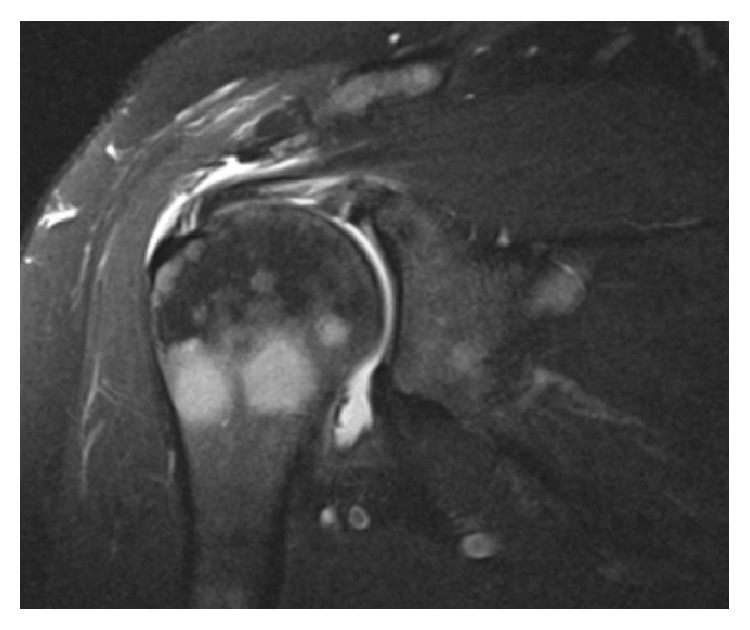
MRI demonstrating sarcoid lesions of the humerus.

Her right shoulder pain worsened acutely in April 2016 after her dog jerked on the leash, almost causing her to fall. Radiographs obtained one month later demonstrated a hooked acromion and subtle decrease in trabecular bone density adjacent to the greater tuberosity, but no distinct bone lesions were noted (Figure 1). The subtle decrease in trabecular bone was considered to possibly reflect disuse osteopenia associated with a long-standing rotator cuff tear [16–18]. Subsequent MRI with intra-articular contrast was obtained which demonstrated what was interpreted as a small full-thickness tear of the supraspinatus tendon. The MR images also revealed multiple quasi-circular lesions within the proximal humerus that were suggestive of metastases or multiple myeloma (Figure 2). Bone lesions were also in close proximity to the insertion of the supraspinatus tendon.

**Figure 3:**
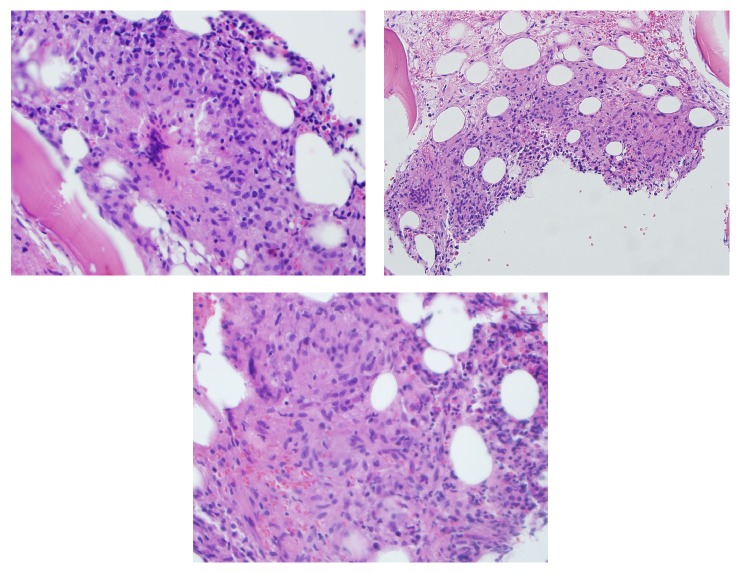
Biopsy of humeral lesions demonstrating noncaseating granulomas.

Ten days prior to the radiographs, she had an unrelated skin biopsy of a facial lesion that was diagnosed as sarcoidosis. A biopsy of the humeral lesions seen on MRI revealed noncaseating granulomatous inflammation, confirming osseous sarcoid of the humerus (Figure 3). Additionally, mediastinal and hilar adenopathy seen on a subsequent chest computed tomography (CT) were consistent with the diagnosis of sarcoidosis.

With osseous sarcoid lesions, there is concern that pathologic disturbances in bone architecture could make surgical repair involving cortical bone difficult [19]. For example, Ungprasert et al. [20] reported an increased incidence of fragility fracture in the proximal humerus in patients with sarcoidosis of the humerus. However, given that our patient's humeral lesions involved cancellous bone, while cortical bone was spared, we concluded that the lesions were not a contraindication to a standard surgical repair of the rotator cuff tear. This is because only a small amount of cortical bone needs to be removed during the repair of a small rotator cuff tear [18, 21]. However, larger tears and/or cases with more invasive sarcoid lesions might require advanced repair techniques [22, 23]. This was not a concern in our patient's case because during surgery only a low-grade partial tear was found. Surgical treatment was done by JGS and included arthroscopic debridement, bursectomy, and acromioplasty [24, 25]. The shavings from the surgical procedure were not obtained for histological analysis. However, the tissues did not appear to be grossly abnormal.

Despite being enrolled in a physical therapy program at two weeks after surgery, she had an unusually prolonged recovery because of flares of pain in addition to shoulder stiffness. Treatment for this included a one-time seven-day course of an oral corticosteroid on a tapered dose schedule (methylprednisolone tablets; Medrol® dose pack; Pfizer Inc., New York, USA). Because the improvement with this was less than satisfactory, two sets of subacromial and glenohumeral corticosteroid injections (Depo-Medrol injectable suspension; Pfizer Inc., New York, USA) were administered two months apart [26]. Supervised physical therapy was also continued.

Because of continuing shoulder and generalized pain flares coupled with a great sense of fatigue, the patient consulted with a rheumatologist who prescribed adalimumab (Humira®, AbbVie Inc., North Chicago, Illinois, USA) subcutaneous injections once every other week. Adalimumab is a tumor necrosis factor-alpha (TNF-*α*) inhibitor that was selected over other first-line therapies for sarcoidosis because of its greater effect on sarcoidosis-associated fatigue [27]. Our patient reported that the adalimumab injections greatly improved her fatigue and thereby enhanced her ability to participate in physical therapy. At final postoperative follow-up at 48 months after her shoulder surgery the patient reported that she had an excellent result that included complete pain relief and restoration of full range of motion and strength.

## 3. Discussion

Sarcoidosis of the humerus is exceptionally rare, and few cases have ever been reported. To our knowledge, sarcoidosis of the humerus with a coexisting rotator cuff tear has not been reported. Importantly for this case, osseous sarcoidosis can compromise normal bone architecture and lead to pathologic fractures, thus potentially complicating surgical procedures [19]. However, our patient had a successful surgical debridement of her rotator cuff tear despite bony involvement of her sarcoidosis.

One of the challenges in diagnosing osseous sarcoidosis is that while bony lesions may cause pain, most patients are asymptomatic [8, 19]. Whether or not our patient's shoulder pain was in fact due mostly to subacromial bursitis or underlying sarcoidosis, or a combination of the two, is uncertain. Another challenge in the diagnosis of osseous sarcoidosis is that these lesions might not appear on radiographs, and more sensitive imaging modalities like MRI are needed to make the diagnosis. MRI of sarcoidosis in long bones typically demonstrates abnormal foci of low signal intensity on T1-weighted images and high signal intensity on T2-weighted images [7]. Involvement of cortical bone is less likely in these long bone lesions [7, 14, 19]. Lack of cortical involvement may explain why many sarcoid lesions remain undiagnosed with radiographs in most cases. Mehrotra et al. [13] reported the case of a 59-year-old woman with sarcoidosis of the humerus that appeared as lytic lesions on radiographs. However, detection on radiographs is the exception, and MRI appears to be the most sensitive modality for detecting bone involvement [9].

Chaudhry and Richardson [7] described a case of a 50-year-old male with a history of pulmonary sarcoidosis who had increased uptake in his upper right humerus on a 99mTc radionuclide scan. An initial radiograph of the shoulder was read as normal. MRI showed an abnormal lesion in the proximal medial humeral head. A biopsy revealed noncaseating granulomas, confirming osseous sarcoidosis. This patient had previously developed psychotic side effects from corticosteroid therapy (route of administration not stated) and was therefore treated with radiotherapy. His symptoms later improved. He did not have a rotator cuff tear or other shoulder pathology, and he did not have shoulder surgery (other than the biopsy).

Yachoui et al. [15] reported the case of a 58-year-old Caucasian woman who initially presented with intermittent shoulder pain that eventually became constant. A plain radiograph of the shoulder was normal. However, MRI demonstrated irregular signal in the upper humeral metaphysis. A subsequent CT scan demonstrated hilar and mediastinal adenopathy. Positron emission tomography (PET) with 18F-fluorodeoxyglucose (18F-FDG) showed numerous areas of increased activity, which was suspicious for metastatic cancer. Histological examination of one of the lesions revealed small granulomas with epithelioid cells, consistent with osseous sarcoidosis. Their patient improved after a course of prednisone and hydroxychloroquine.

Similar to these cases, our patient's radiograph was unremarkable, and follow-up MRI showed multiple lytic bone lesions of the humerus that were concerning for metastases or multiple myeloma, as sarcoidosis can mimic these conditions [5, 8–12]. Additionally, biopsy in each case revealed histological findings consistent with sarcoidosis. These, as well as additional cases, are compared in Table 1.

## 4. Conclusion

In summary, we report a rare case of sarcoidosis of the upper humerus. The novel aspect of our case was that sarcoid lesions were found incidentally with MR images obtained to evaluate the patient's shoulder for a rotator cuff tendon tear. Notably, the integrity of the bone was not compromised to the point that surgery could not be performed, and our patient had arthroscopic debridement of a concurrent partial rotator cuff tear. The presence and locations of the sarcoid lesions did not influence the surgical procedure because they did not involve the cortical bone and tendon repair was not needed because the tear was partial. The excellent outcome that was ultimately achieved was facilitated by prolonged physical therapy and adalimumab injections. Follow-up at 48 months demonstrated full recovery from her rotator cuff tear and no residual symptoms in her shoulder.

## Figures and Tables

**Table 1:** Radiological findings of sarcoid of the humerus.

Authors	Patient demographics	Presentation	Past medical history	Radiological findings	Diagnosis	Management
Mehrotra et al. 2011	59-year-old woman	Left facial palsy after coryzal illness, otherwise asymptomatic	Facial nerve palsy	Radiographs: lytic lesions in the humerus CT thorax/abdomen: low attenuation liver lesions and widespread vertebral, rib, pelvic lytic lesions	Biopsy	Did not receive steroids, has been stable for one year. Still has facial palsy.

Chaudhry and Richardson 2006	50-year-old man	Two-month history of right shoulder pain and mildly decreased mobility	Pulmonary sarcoidosis for eight years	Radiograph: faint sclerotic lesion in the inferomedial humeral head MRI: abnormal marrow signal intensity in the medial humeral head. Low signal intensity on both T1- and T2-weighted images. STIR image demonstrated a hyperintense signal focus within the marrow without cortical involvement CT: focal area of mild sclerosis in the inferomedial humeral head 99mTc radionuclide scan: mild uptake in the humeral head	Biopsy	Did not receive steroids (history of psychiatric side effects) and was treated with radiotherapy. His symptoms later improved.

Yachoui et al. 2015	58-year-old Caucasian woman	Six months of intermittent shoulder pain that eventually became constant	Hypertension, tonsillectomy	Radiograph: normal MRI: high signal intensity on T2 fat suppressed sequence, intermediate intensity on T1-weighted imaging with postcontrast enhancement in humeral metaphysis CT: hilar and mediastinal adenopathy PET: numerous areas of increased activity	Biopsy	Their patient improved after a course of prednisone and hydroxychloroquine.

Henrie and Skedros 2018	50-year-old woman	Several-year history of intermittent low-grade right shoulder pain, which worsened acutely after her dog jerked on the leash	Diabetes, hypertension, anxiety, depression, and fatigue	Radiograph: normal MRI: multiple lytic bone lesions of humerus	Biopsy	Did not receive steroids (contraindicated given history of diabetes). Received prolonged physical therapy and adalimumab injections.
